# A Brief Recap of Tips and Surgical Manoeuvres to Enhance Optimal Outcome of Surgically Placed Peritoneal Dialysis Catheters

**DOI:** 10.1155/2012/251584

**Published:** 2012-07-19

**Authors:** Jodie H. Frost, Atul Bagul

**Affiliations:** Transplant Division, III Department, University of Leicester, Leicester LE1 7RH, UK

## Abstract

*Background*. Peritoneal dialysis (PD) is an effective option of renal replacement therapy for ESRF, offering advantages over haemodialysis. Peritoneal dialysis catheter (PDC) placement is thought to be the key to successful PD and the economic advantages are lost if a patient switches to HD in the 1st year. This paper is a brief document elaborating a recap of published literature, looking at various surgical tips and manoeuvres to enhance optimal outcome of PDC placement. *Methods*. A search strategy assessing for access team, preoperative antibiotic prophylaxis, type of catheter, catheter exit site, intraoperative catheter trial, optimal time to commence PD, hernia repairs, number of cuffs, catheter-embedding procedures, rectus sheath tunnelling, laparoscopic fixing, omentopexy, omentectomy, the “Y”-Tec system, resection of epiploic appendages, adhesiolysis, a trained surgeon, and perioperative catheter care protocol was used looking at various databases. *Findings*. The complications of catheterrelated dysfunction can be reduced with advanced planning of access placement, immaculate surgery, and attention to catheter insertion techniques. *Conclusion*. The success of a peritoneal dialysis programme depends upon functional and durable long term access to the peritoneal cavity; this depends on placement techniques and competent surgeons and psychosocial support to the patient. The various technical tips and manoeuvres elaborated here should be considered options carried out to improve outcome and reduce catheter dysfunction.

## 1. Introduction

Peritoneal dialysis (PD) is an effective option of renal replacement therapy for end-stage renal failure, offering advantages such as it being a modality undertaken at home, encourages self-autonomy, and optimises quality of life [1, 2]. It is well established that access-related morbidity and mortality are significantly lower for PD than for Haemodialysis (HD) [3]. Its other advantages are improved survival benefit during the first 1 to 2 years due to better preservation of residual function and improved blood volume and blood pressure control [4, 5]. Peritoneal dialysis is less expensive than HD, a cost difference of $19,000/person/year in the USA (PD8–28) and *£*12,000/person/year in the UK (NHSBT-website: http://www.organdonation.nhs.uk/). The success of PD depends upon the presence of a functional and durable long term access to the peritoneal cavity, which depends upon placement technique rather than on catheter design [6].

Thus, catheter placement is thought to be the key of successful PD and the economic advantages are lost if a patient switches to HD in the 1st year [7]. Primary nonfunction rate for PDC in literature is quoted at 10% [8]. The potential complication for PDC placement includes mechanical flow dysfunction usually associated with malposition, leak, exit-site infection/peritoneal infection, intra-abdominal injuries, and catheter blockages [9]. These complications can be reduced with advanced planning of access placement, immaculate surgery, attention to catheter insertion techniques, and psychosocial support to the patient [10, 11]. Catheter-related problems like recurrent leaks and hernias, leading to a delay for PD and if not further surgical procedures increase pyscho-social problems in this cohort of patients. A multidisciplinary team involving PD nurses, nephrologist, and surgeons will increase the psychosocial support to such a cohort of patients and add to the success of a PD programme.

## 2. Objective

This paper is a brief document elaborating a recap of published literature, looking at various surgical tips and manoeuvres to enhance optimal outcome of peritoneal dialysis catheter placement.

## 3. Search Strategy

Databases: Medline (Pubmed-1966–2011), Cochrane Central Register of Controlled Trials, EMBASE (1974–2011) the Data base of Abstracts of Reviews of Effects (DARE), and Health technology assessment database (HTA).

The search strategy was based on the following:

- peritoneal dialysis catheters inserts,
- peritoneal dialysis catheter dysfunction,
- surgical manoeuvres for peritoneal dialysis,
- laparoscopic peritoneal dialysis catheter insertion,
- open PDC insertions,
- Tenckhoff PDC catheters,
- peritoneoscopic PDC insertions,
- antibiotic prophylaxis for PDC insertion,
- controversy of PDC,
- role of surgeon in PDC placement.

The various tips and surgical manoeuvres to enhance optimal outcome of peritoneal dialysis catheters are discussed as follows.

### 3.1. Peritoneal Access (PD Access): The Access Team

The Renal Association UK: Peritoneal Access Guidelines: guideline peritoneal dialysis (RA/PAG:PD) [12] and International Society for Peritoneal Dialysis (ISPD) guidelines recommend that each centre should have a dedicated team involved in the implantation and care of peritoneal catheters. (RA/PAG: PD1.1) [13] (ISPD: 1.1) [14].

### 3.2. Preoperative Antibiotic Prophylaxis

A single dose of preoperative antibiotics is recommended (based on unit microbiology policy)( RA/PAG: PD3.1) [12] (ISPD:3.1) [14] and this reduces the rate of exit-site infection and peritonitis [9, 15–20]. 

### 3.3. Type of Catheter

 RA/PAG: PD5.2 [12] and ISPD: 5.2 suggest that no particular catheter type is proven to be better than another [21]. Though, a curled tip catheter should be used and is more likely to retain position within the pelvis and offers a better drainage compared to a straight tip catheter [22, 23]. Nielsen et al., published a small RCT which demonstrates superiority of the curled Tenckhoff peritoneal dialysis catheter survival as compared to the straight catheter which was due to the higher displacement rate [24]. In addition, straight tip catheters are associated with dialysate inflow discomfort or the “poking pain” which is not reported with curled catheters [22]. An extended catheter system or two-piece extended catheter system can be used to provide remote exit-site locations to the upper abdomen or chest as an indication for obesity, floppy abdominal skin folds, presence of stomas, incontinence, and yeast intertrigo [25–28]. Self locating catheters have been described, which show a lower rate of displacements [29–32].

### 3.4. Catheter Exit-Site

The exit-site should be well planned, ideally on the opposite side of the dominant hand and should not be placed in skin crease, belt line and should be located dependently to the site of cuff placement. (RA/PAG) Many authors have described optimum exit-sites in literature, yet, the ideal situation is that the patient should be marked in a preoperative setting. If this has not been done, then the 3-step algorithm method could be used to determine exit-site at the time of the operation [10]. It is to design a laterally directed exit-site that places the superficial cuff 4 cm from the exit wound and also eliminates concerns for cuff extrusion from tubing resiliency forces. In brief, the three steps include the following Step 1: scribe arc from vertical to lateral plane using catheter as compass from point 2 cm external of superficial cuff. Step 2: mark exit-site at junction of medial 2/3rd and lateral 1/3rd of arc. Step 3: indicate tunnel tract shape by bending catheter over from point 4 cm external of superficial cuff to exit-site.

### 3.5. Intraoperative Catheter Trial

Various volumes have been used but we prefer to use half a bag of PD fluid to check for instillation and drainage. This is a very important and crucial step as this manoeuvre does predict successful function (inflow and outflow) of the PDC postoperatively. In addition, further small volume exchanges can be carried out in patients who have undergone extensive adhesiolysis until draining dialysate is no longer blood stained [9]. In addition, it should also be used to test the tightness of catheter at the peritoneum, thus excluding a possible dialysate leak.

### 3.6. Optimal Time to Commence PD

An approximate 2-week delay (RA/PAG: PD2.1) [12] (ISPD: 2.1) [14] results in the less likelihood of exit-site infection and dialysate leak [9, 33], though this is based on evidence from mostly retrospective studies. Reported leak rates are significantly lower for all documented laparoscopic series, even when catheters are used straight away [1, 34–38]. Purse string sutures at the cuff to anchor have been described during open surgery and do reduce the risk of early leaks [39, 40]. Yang et al., did not show higher risk of catheter-related problems in PD patients starting dialysis less than 2 weeks (earlier group (2.0 ± 2.7 days) versus late group (40.6 ± 42.8 days): 14.6% and 13.1%, resp. developed catheter-related complications within 6 months) [41]. Currently, a randomised control study (timely PD study) has been set up to address the influence of different break in periods [42].

### 3.7. Hernia Repairs

Utmost importance should be given to the consent, where the patient should be counselled for hernia repair found at the time of the operation. Gracía-Ureña et al. in their article yet stress the importance of identifying and repairing of hernias prior to commencing PD [43]. The hernioplasty should be performed using prosthetic material as a tension-free repair, hence, to reduce the risk of recurrence in peritoneal dialysis patient [43–45]. This may lead to a small draw back being a delay in initial catheter use for peritoneal dialysis and patients should be warned about interim haemodialysis [9, 10], though literature supports successful immediate restart of PD [45].

### 3.8. Number of Cuffs

Controversy lies with either using a catheter with a single or two cuffs. A single-cuffed catheter logically avoids a second foreign body being placed in the less-vascularised subcutaneous tissue, thereby reducing the risk of cuff infections or colonisation near the exit-site [9]. Eklund et al., published in a small RCT (30 per arm) that there to be no significant difference between catheters with single or double cuffs with respect to catheter survival, episodes of peritonitis, and exit-site infections [46]. Though, some published lines of literature prefer two-cuffed catheters as they reduce risk of peritonitis in the long run [9]. Nessim et al., reported double cuff were associated with reduced peritonitis rates between 1996 and 2000, though after 2000 this risk disappeared and was probably due to influence of prophylactic measures or increase in experience on the risk of infection over time [47]. The Cochrane Review did not find any advantage for straight versus coiled catheters, single or double cuff, and median or lateral incision [21] and the ISPD guidelines recommend no difference (ISPD: 5.2) [14].

### 3.9. Catheter-Embedding Procedures

These procedures were initially described by Moncrief et al., [48], where the procedure entails the external limb of the dialysis catheter is embedded subcutaneously at the time of implantation. After about 3–5 weeks interval, exteriorization is carried out under local anaesthesia in the day case setting. Brown et al., recommend insertion 6 weeks to 5 months ahead of the need for PD to maximize catheter survival. The authors looked at three groups of patients starting PD 11–47 days, 48–133 days, and more than 133 days after placement of embedded catheters. The time to catheter loss was shortest in group 1 and longest in group 2 and suggested that mechanical complications and catheter loss are associated with the length of time a catheter is embedded [49]. The concept speculated reduced catheter-related infection due to firm tissue ingrowth of the cuff and the absence of a biofilm. With time and use of antibiotic prophylaxis these procedures had initially lost their popularity. But in reality it may be a useful strategy to lock a patient into peritoneal so as to avoid crash haemodialysis with a tunnelled catheter if sudden decline in renal function were to occur. The technique of embedding allows efficient surgical scheduling of catheter placement as a planned procedure which can lead to a significant longer catheter life [50] and fewer earlier complications [51]. 

### 3.10. Rectus Sheath Tunnelling

This leads to creation of a long musculofascial tunnel in a craniocaudal direction, thus maintaining pelvic orientation of the catheter tip [52–55]. This manoeuvre leads to reduced risk of pericannular leaks and hernias [10]. A detailed step-by-step technique for rectus sheath tunnelling has been described by Crabtree [10]. To facilitate a further reduction of leaks during transrectus placement, a fixation suture around the catheter at the anterior rectus sheath may be placed, with the cuff being secured at this fascial layer [56–58]. Attaluri et al., data clearly showed a significant improvement in PD catheter function using omentopexy and rectus sheath tunnelling [58].

### 3.11. Laparoscopic Fixation

Laparoscopic fixation to prevent catheter tip migration may be carried out where the catheter tip is fixed to the lower abdomen or pelvis [59, 60]. The drawback here is an increased risk of obstructing adhesions, potential for internal hernias and suture knot, a nidus for persistent infection; proper rectus sheath tunnelling and placement of the deep cuff are the key to reducing catheter tip migration [10].

### 3.12. Omentopexy

Omentopexy is usually carried out when partial or complete catheter obstruction/displacement is produced due to omental entrapment. The procedure involves a laparoscopic omental tack up, which is performed high in the abdomen at the Palmer point [61] or to the falciform ligament [62]. This procedure is not limited to rescuing catheters from omental entrapment and does play a valuable role in selective prophylactic omentopexy as an adjunctive procedure to catheter implantation as assessed by an intra-operative catheter trial [6] and especially when the omentum extends to the pelvis in a juxtaposition of the peritoneal catheter tip [63].

### 3.13. Omentectomy

Omentectomy is usually avoided as an omentopexy is a much quicker and cost-effective procedure with an equivalent outcome [10, 64]. Persistent dysfunction problems due to omental wraps and failed omentopexy warrant an omentectomy though rare. 

### 3.14. The Modified “Y”-Tec System

This system permits the procedure to be carried out with either a single 5 mm lap-port or a 2.2 mm peritoneoscope, thus leading to reduced incisions, leaks, and hernias. In addition, in particular with the laparoscope, an adequate and thorough diagnostic look can be carried out in particular for redo/exchange of PD catheters. Additional ports can be inserted to carry out procedure like adhesiolysis, omentopexy, and peritoneal biopsies [65].

### 3.15. Resection of Epiploic Appendages

Epiploic appendages are an infrequent cause of catheter dysfunction and can be identified during the intraoperative catheter trial irrigation test. Once identified the offending epiploic (seen blocking the catheter during an outflow check) appendages indicate a laparoscopic resection [66].

### 3.16. Adhesiolysis

Adhesions play a significant role in catheter displacement, thus dysfunction. Extensive dissection is neither necessary nor desirable to mobilise and divide every single adhesion. Abdominal wall adhesion above the level of the pelvis will not interfere and is best left alone. Laparoscopic dissection can be carried out using either scissor with diathermy, hook diathermy or ultrasonic shears. Limiting adhesiolysis to that necessary avoids visceral injury, haemorrhage, and reformation of adhesions which will eventually compromise the peritoneal cavity [10].

### 3.17. A Trained Surgeon

Surgical outcomes strongly corelate with training during residency [67]. RA/PAG PD6.1 [12] recommend that PD catheter insertion training should be available to all trainees with an interest [68]. Placement of a PDC by an experienced surgeon is strongly recommended to reduce complications [69]. Repetition of the procedure is needed for surgical proficiency [67, 70] and the number quoted is 20–40 procedures before an acceptable level of skill can be obtained [70, 71]. RA/PAG PD6.2 [12] recommend that PD catheter insertion should not be delegated to inexperienced unsupervised operators [13].

### 3.18. Patient Selection

Surgical placement of PDC should be the standard for complicated patients such as PDC revisions and patients with hernias and redo abdominal surgery. The procedure should be carried out as a laparoscopic placement (direct visualisation; ISPD guideline 4.1) under a general anaesthesia as problems such as adhesions and hernias can be dealt with at the time of surgery [10, 15, 28, 61, 65]. The anaesthetic requirement depends on the technique selected, which is influenced by the patients characteristics (ISPD: 5.1) [14] and thus for complicated patients general anaesthesia is preferred.

ISPD guideline 4.1 recommend that local expertise at individual centres should govern the choice of method of PDC placement, with timely surgical support available for the review of PD patients (ISPD: 4.4) [14]. There is no RCT evidence to support one method of insertion over another; however, the method needs be determined by patient characteristics (ISPD: 4.4) [14]. Indeed, some data show that laparoscopic PDC implantation may be superior to other methods in patients with pervious operation [58, 72], though results of the RCT LOCI trial are awaited [73].

### 3.19. Perioperative Catheter Care Protocol

RA/PAG PD 3.1 and ISPD 3.1 recommend that renal units should have clear protocols for perioperative catheter care addressing the following [12, 15]:

- Preoperatively—check for hernias, screen for MRSA, and nasal carriage of *Staphylococcus aureus*, identifying a catheter of a suitable length, and mark the appropriate exit-site sitting and standing.
- Before-implantation—bowel preparation with laxatives, ensuring bladder emptying, administration of prophylactic antibiotics, and surgical site preparation according to NICE guidance [18].
- Aftert-procedure—catheter flush and cap off using suitable dialysate, exit-site covered with a suitable nonocclusive dressing and if possible not disturbed for 5–10 days, immobilisation of the catheter, patient discharged home with supply of aperients with advice as how to recognise potential complications. Intraperitoneal administration of heparin catheter flushes prevents fibrin formation in CAPD patients and may have added benefits [74].

Once the catheter is placed and until healing is completed, the dressing changes should be addressed by a dialysis follow-up nurse using sterile technique.

### 3.20. Contraindications for PDC (http://www.kidney.org/professionals/kdoqi/guidelines/)

Absolute contraindications for PD include (guideline 30: NKF KDOQI guidelines for peritoneal dialysis adequacy)

- documented loss of peritoneal function or extensive abdominal adhesions that limit dialysate flow,
- uncorrectable mechanical defects that prevent effective PD or increase the risk of infection (e.g., surgically irreparable hernia, omphalocele, gastroschisis, diaphragmatic hernia, and bladder exstrophy).

Relative contraindications for PD include the following. (Guideline 31: NKF KDOQI).

- Fresh intra-abdominal foreign bodies (e.g., 4-month wait after abdominal vascular prostheses, recent ventricular-peritoneal shunt).
- Peritoneal leaks.
- Body size limitations.
- Intolerance to PD volumes necessary to achieve adequate PD dose.
- Inflammatory or ischemic bowel disease.
- Abdominal wall or skin infection.
- Morbid obesity (in short individuals).
- Severe malnutrition.
- Frequent episodes of diverticulitis.

## 4. Conclusions

The success of a peritoneal dialysis programme depends upon functional and durable long term access to the peritoneal cavity; this depends on placement techniques and competent surgeons. The various technical tips and manoeuvres elaborated here should be considered options carried out to improve outcome and reduce catheter dysfunction.
